# Addressing metastatic individuals everyday: Rationale and design of the nurse AMIE for Amazon Echo Show trial among metastatic breast cancer patients

**DOI:** 10.1016/j.conctc.2023.101058

**Published:** 2023-01-16

**Authors:** Brett R. Gordon, Ling Qiu, Shawna E. Doerksen, Bethany Kanski, Abigail Lorenzo, Cristina I. Truica, Monali Vasekar, Ming Wang, Renate M. Winkels, Saeed Abdullah, Kathryn H. Schmitz

**Affiliations:** a Pennylvania State University College of Medicine, Hershey, PA, USA; b Pennsylvania State University College of Information and Science Technology, University Park, PA, USA; c Division of Human Nutrition and Health, Wageningen University, the Netherlands

**Keywords:** Digital health interventions, Breast neoplasms, Metastases

## Abstract

**Background:**

Metastatic Breast Cancer (MBC) patients often feel their symptom-related needs are unmet, despite visiting their doctors up to once a week. Novel approaches are needed to address symptoms without requiring additional appointments. Technology based symptom management approaches to address symptoms have not been well tested.

**Methods:**

Nurse AMIE (Addressing Metastatic Individuals Everyday) is a technology based supportive care platform that provides guideline-concordant symptom management interventions in response to patient reported symptoms. We have previously successfully implemented a tablet version of Nurse AMIE. However, some eligible patients chose not to participate because they were overwhelmed by the technology. To address this barrier, we translated the Nurse AMIE platform to the Amazon Echo Show, which allowed for voice-based interactions. Forty-two MBC patients were randomized 1:1 to receive the Nurse AMIE for Echo Show immediately for six months, or to receive the same intervention for three months, after a three month delay. The primary outcome was change in physical distress over three months, and secondary outcomes included feasibility, acceptability, patient reported outcomes and usability.

**Conclusions:**

Results from the Nurse AMIE for Echo Show trial will identify the feasibility, acceptability, and preliminary effects of the Nurse AMIE for Echo Show on patient reported outcomes. Untested novel technologies, particularly voice-based artificial intelligence devices may an effective and scalable vehicle through which we can deliver supportive care interventions.

**Clinicaltrials.gov identifier:**

NCT04673019.

## Highlights

• Metastatic Breast Cancer patients often feel their symptoms-related needs are unmet.
• Nurse AMIE for Echo Show provides guidelines-concordant interventions for symptoms.
• Untested novel technologies may be an effective an scalable approach to deliver care.

## 1 Introduction

Improvements in treatments over the past decade have led to a growing number of metastatic breast cancer (MBC) patients [1]. It is estimated that more than 150,000 women in the United States are living with MBC [2]. Recent research has indicated MBC patients often feel their symptom related needs are unmet, and MBC patients have expressed desire to address these issues to improve their quality of life [3]. MBC patients also expressed that methods to cope with side-effects and symptoms are important informational needs [4,5]. For some MBC patients, the severe side-effects of their treatments reduce quality of life to such an extent that they make decisions to stop treatment, and allow nature to take its course, with a high likelihood of mortality. In this sense, mitigation of side-effects may have survival benefits.

Although MBC patients express desire for more help with symptoms, increased visits to the treatment center can be burdensome; some MBC patients may already have medical appointments as often as once a week [6]. Approaches are required to address symptom needs without additional appointments. Additionally, prior research indicated patients desired a nutrition component be added to Nurse AMIE [7]. Technology may be a useful tool to assess and address the needs of MBC patients, including options to enhance patient-provider communication, and provide guideline-concordant self-care interventions to reduce symptom burden [8]. These technologies take many shapes and forms, and the best ways to use technology to serve the needs of MBC patients are unclear. In prior research, we developed a tablet-based supportive care platform called Nurse AMIE (Addressing Metastatic Individuals Everyday) [8]. It was observed to be feasible and acceptable; patients reported liking the interventions offered on the tablet 83% of the time [8]. However, in this prior work, some MBC patients we approached for the Nurse AMIE intervention declined to participate because they felt overwhelmed by the tablet technology.

To address these issues, we worked with human-computer interaction (HCI) researchers (SA and LQ) to translate the Nurse AMIE intervention to the Amazon Echo Show, a voice activated smart speaker and display device. We also worked with a nutrition researcher (RW) to add nutrition education and support to Nurse AMIE. In this manuscript, we present the development and rationale of this revised Nurse AMIE intervention. The Nurse AMIE for Amazon Echo Show trial randomized 42 MBC patients to either an immediate start (immediate AMIE) or delayed start (delayed AMIE). Hypotheses of this research were: 1) Nurse AMIE on the 10.13039/100016443Amazon Echo Show will demonstrate equal or better acceptability as compared to the tablet only version, which showed 68% acceptability, 2) Nurse AMIE on the 10.13039/100016443Amazon Echo Show will have at least 86% feasibility (defined as at least 30 days of use out of 90 possible days), and 3) using the Nurse AMIE for 10.13039/100016443Amazon Echo Show will improve physical distress (primary outcome), as well as physical function, health related quality of life, sleep, emotional support, pain, and fatigue. After funding, we added surveys to more robustly assess usability of Nurse AMIE on the 10.13039/100016443Amazon Echo Show.

## 2 Methods

This manuscript adheres to the Standard Protocol Items: Recommendations for Interventional Trials (SPIRIT) Checklist [9]. This investigation was carried out in accordance with the latest version of the Declaration of Helsinki. This study protocol was approved by the Penn State University Institutional Review Board (STUDY00016221).

### Support

Funding for this trial was provided by the 10.13039/100000969American Institute for Cancer Research. The funding body played no role in the study design, methods, participant recruitment, data collection, analyses, or preparation of this manuscript.

### 2.1 Eligibility criteria

Potential participants were included if they met the following criteria: they were a female patient with MBC, age ≥18 years, had personal, in-home Wi-Fi access, a personal device capable of participating in Zoom calls, fluency in written and spoken English, and sufficient vision/hearing to use the device. Participants were excluded if they had an Eastern Cooperative Oncology Group (ECOG) performance status score >2, significant medical or psychiatric conditions (beyond breast cancer, its treatment, and its symptoms), were receiving any behavioral intervention, were pregnant, had cognitive impairment, or had a life expectancy of less than six months as determined by the treating physician. As MBC patients' conditions are subject to change over time, we sought a second approval from participants’ treating oncologist in the delayed AMIE condition before they started Nurse AMIE to ensure their inclusion/exclusion criteria had not changed during the study. If their treating oncologist indicated they were no longer eligible, we would call the participant and explained this situation to them.

### 2.2 Recruitment

Participants were recruited by scanning the electronic medical record of patients seen in the cancer institute to identify potentially eligible women with MBC. Participants were also recruited through social media outreach. The patient's oncologist and/or primary physician provided pre-approval for participants identified from any source. Oncologists at the College of Medicine were also aware of the study, and referred eligible participants to our study staff. We also used a web-based recruitment tool for Penn State researchers, “Penn State Studyfinder” (available at: https://studyfinder.psu.edu), and shared information with national organizations that serve the needs of MBC patients (BreastCancer.org and the Metastatic Breast Cancer Coalition). Research staff approached potentially eligible participants over the phone.

### 2.3 Study design

**Fig. 1:**
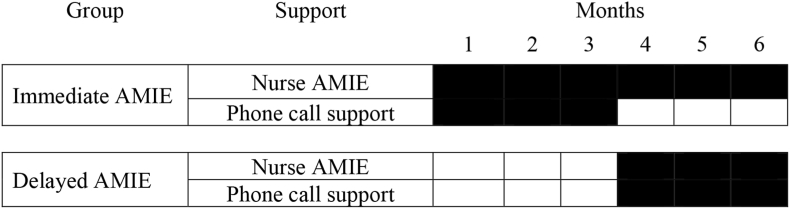
Description of nurse AMIE (Alexa echo show) design.

The Nurse AMIE for Amazon Echo Show trial was designed as a partial crossover pilot study with two arms: (1) immediate AMIE and (2) delayed AMIE. The immediate AMIE group received Nurse AMIE for a total of six months. For the first three months, the immediate AMIE group received weekly phone call support from a study facilitator as part of Nurse AMIE. The delayed AMIE group received Nurse AMIE in months four through six only, which included weekly phone call support (Fig. 1).

**Table:** 

Group	Support	Months					
		1	2	3	4	5	6
Immediate AMIE	Nurse AMIE						
	Phone call support						

Delayed AMIE	Nurse AMIE						
	Phone call support

### 2.4 Randomization and blinding

During baseline assessments, participants were randomized using a random sequence generated by a researcher not involved in data collection. Conditions (Immediate AMIE or Delayed AMIE) were blindly placed in sealed envelopes, and not opened until baseline assessments were completed. Randomization was not stratified. Participants were not blinded to their group assignment. Study team members involved in patient-facing work, such as the study-facilitator calls, were also not blinded to group assignment. The principal investigator was blinded to participant allocation, and all study analyses will be performed by study team members blinded to group status.

### 2.5 Translating the tablet-based nurse AMIE to the Amazon Echo Show

The Amazon Alexa ecosystem uses audio processing and machine learning for understanding voice commands and inputs from users. It also provides an Application Programming Interface (API), which can be used to develop a highly customized and context-aware dialogue system (“skill”). In this project, we leveraged the API to develop Nurse AMIE as an Alexa skill. More specifically, we provided the following functionalities to MBC patients: i) voice interface; ii) dynamic information delivery; iii) collection of data from patients (e.g., step counts; symptoms); and iv) sharing data with facilitators and clinicians. We describe these functionalities in detail below.

### 2.6 Voice interface

Voice interface allowed users to interact with the device using just speech and audio commands. Voice interfaces enable interactive, engaging, and dynamic delivery of content. To develop the Nurse AMIE for Amazon Echo Show voice interface, we adapted the existing content from the tablet version of Nurse AMIE. This involved determining message content, identifying turn-taking points, and establishing potential branching based on inputs from a participant. We also focused on smart and dynamic handling of conversations to adapt to different user-initiated scenarios and contexts. This resulted in a personalized “virtual coach,” that reflected participants’ unique needs and interaction styles. The user speaks to the Echo, says “Open Nurse AMIE,” and the program opens. The screen depicts a nurse in a lab coat and Nurse AMIE verbally greets the participant and provides a nutrition tip. Nurse AMIE then asks the participant symptom questions. The participant answers, just like she is speaking with a human nurse. Based on the answers the participant provides, Nurse AMIE gives an intervention to help those symptoms.

To develop the voice interface, we adopted an iterative process. In the first phase of this process, we developed a set of decision trees (“flow diagrams”). In these decision trees, branches indicate different interaction pathways following user responses. These decision trees provided the blueprint for user interface and navigation in the Alexa skill. During the development phase, we followed established design guidelines for effective voice interactions (e.g., supporting quick interaction turns, and being adaptable). We also conducted in-lab usability tests with small samples of patients and stakeholders to ensure the voice interface is robust, easy to navigate, and highly useable [10].

### 2.7 Dynamic information delivery

**Fig. 2:**
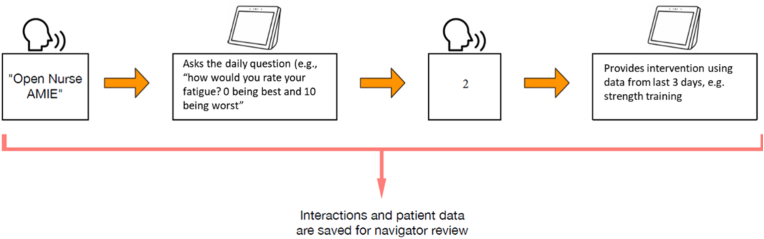
Nurse AMIE assesses symptoms daily to sustain patient engagement and guidelines-concordant interventions.

Dynamic content changes based on the behavior, preferences, and interests of a user. The dynamic content is personalized and adapts based on the data from the user. The goal of the dynamic information delivery is to provide an engaging and satisfying experience. Dynamic information delivery is generally powered by applications and scripts, working in tandem with static content. Examples relevant to the Nurse AMIE Alexa Skill might be responses to fatigue (Fig. 2). Fatigue is a common issue in MBC patients. To address fatigue, the Alexa skill could provide dynamic guidance. For example, participants were asked about their fatigue level every day as one of the daily questions (other questions probed sleep, distress, and pain). In response to high fatigue, patients were provided with walking step goals and exercise videos. Participants could use audio commands to start these services at any time, without waiting for the daily greeting and daily symptom question. Furthermore, the skill provided a variety of services, including video interventions aiming to help with coping with symptoms. The skill also provided guided relaxation recordings and soothing music.

### 2.8 Collecting data from patients

In a previous study in which we used a tablet-version of Nurse AMIE, the device allowed patients to record relevant data including daily symptom questions (fatigue, sleep, distress, and pain) and step counts. The tablet version also recorded the use of each of the intervention modules, and time spent within a given module. The Nurse AMIE for Amazon Echo Show extended this functionality so patients could record data using the voice interface. Nurse AMIE would ask how many steps the participant took, and the participant would say the number to enter it in. Using a voice interface could lower participant burden, and streamline the data collection process. Furthermore, collected data could be used by the Nurse AMIE skill to dynamically recommend relevant interventions. For example, if a patient indicated high levels of distress, the skill could suggest an appropriate intervention for coping with symptoms, such as soothing music. We also incorporated questions from the Patient-Generated Subjective Global Assessment (PG-SGA) short form into Nurse AMIE; the full survey was asked monthly [11,12].

### 2.9 Sharing data with facilitators and clinicians

The patient data collected by Nurse AMIE were automatically uploaded to an investigator-facing dashboard. As with the tablet version, the data were reviewed daily by study facilitators, discussed with patients in weekly calls, and could be shared with clinicians through the electronic medical record when elevated symptoms were reported. Discussion with study clinicians led us to set a threshold of 7 or more out of 10 to define ‘elevated symptoms’. Elevated levels of pain or distress were relayed to the patient's oncologist at the time of patients reporting.

### 2.10 Adding nutrition education and support to nurse AMIE

The overall goal of the new nutrition module was to provide evidence-based information about nutrition to MBC patients. The nutrition advice provided was largely based on American Institute for Cancer Research materials [13]. The module included healthy recipes in the pink ribbon menu on the device, and daily nutritional strategies. Nutrition strategies included tips to deal with side effects of cancer and its treatment, tips regarding medications/cancer treatment and possible interactions with nutrition, and tips providing information on weight monitoring, appetite and weight loss. This nutrition content was developed based on the needs that MBC patients have reported previously [7].

We worked as a team to discern whether it was possible to develop a question and answer section where MBC patients could pose questions on nutrition, which would either be answered directly by the Alexa system, or Alexa would “forward” the questions to the facilitators. Based on our initial exploration, it was not feasible to develop this section within our timeline, and we will need to wait for future funding opportunities to implement this section.

### 2.11 Intervention delivery: nurse AMIE for Amazon Echo Show

Participants were provided an Amazon Echo Show, along with a pedometer, set of resistance bands, and participant binder. Materials were shipped to participants, and participants engaged in an orientation session once they received these materials. The participant binder included care team and palliative care team information, instructions for the Amazon Echo Show and Nurse AMIE, instructions for the pedometer, step log sheets, and paper copies of instructions on the exercises. Instructions for exercises were also included as videos in the Nurse AMIE skill.

Exercise content was customized during onboarding based on the participant's metastases location. For example, if there were no bone metastases, there was a three-level exercise program that included upper and lower body exercises that can be done standing. For patients with bone metastases, the exercise programs were more restrictive. For participants with lower extremity bone metastases, there was a chair exercise program only. For participants with spinal metastases, there was a three-level program that avoids spinal twisting motions. For participants with upper body bone metastases, there was a three-level program that avoided stressing the arms and upper torso.

### 2.12 Protection of confidentiality

Two actions were taken to protect confidentiality of the participants while using the Alexa Echo Show. First, no participant provided their actual names or identifying information within the Nurse AMIE platform. Additionally, the participants were instructed to go through the settings in the Amazon Alexa Echo Show device and select the option to ensure no voice recordings were made. Participants were given an alternate email address and identification to be used within the Nurse AMIE for Amazon Echo Show platform. Study team members created the accounts and provided the information to participants.

### 2.13 Daily interactions with nurse AMIE for Amazon Echo Show

To start an interaction with Nurse AMIE, the participant would say “Alexa, Open Nurse AMIE.” The opening screen depicted a nurse waving hello, with a unique spoken greeting for each day of participation. Following the greeting, a nutrition message appeared on the screen, and Nurse AMIE read it aloud to the participant. An external link to a recipe accompanied the nutrition tip, and the participant had the option to visit the recipe at that time, or later in the pink ribbon menu. All of the nutrition education messages, along with links to the informational websites, and healthy recipes presented in categories (e.g., breakfasts, soups and salads, vegetarian) were included in the participant binder. Immediately following the nutrition message, the participant answered the daily symptom questions (rating pain, sleep, fatigue, and distress) on a 0–10 scale. If the answer to the questions about pain or distress were above the *a priori* set clinical threshold of 7 out of 10, the study facilitator contacted the clinical medical team to alert them of the participant's response.

Data from the daily symptom questions fed into a decision algorithm, which determined which type of self-care intervention to offer the participants. Once offered, participants then verbally indicated wanting or not wanting to access that intervention. The algorithm was developed for the tablet-based version of Nurse AMIE, and not changed for the Alex Echo Show version. If participants declined the intervention, they were reminded they can choose their own intervention verbally by instructing Nurse AMIE to “open the pink ribbon menu,” and select their preferred intervention. The Nurse AMIE interventions include exercise videos (strength, stretching, and balance), symptom management education (cognitive behavioral therapy modules), soothing music, guided relaxation, and recipes.

### 2.14 Dashboard and study facilitator calls

An investigator-facing dashboard was developed for Nurse AMIE that allowed study facilitators to review the symptom responses, and which Nurse AMIE interventions were used by participants daily. While receiving Nurse AMIE, the study facilitator called the participant weekly. These weekly calls were set up on a day and time of the participants' choosing. The study facilitator asked them how they were feeling, reviewed elevated symptom responses, and asked if they were having any difficulties or questions about the program. The facilitator also reviewed lifestyle activities the participants were using to manage their symptoms. Additionally, the facilitator provided the participants with an updated individualized step goal based on the steps the participants had recorded using the Nurse AMIE platform. The facilitator averaged the daily steps over the previous week, and set the step goal as that value plus 500 steps. At the end of each week, participants were asked to complete a more in-depth symptom report, the PG-SGA Questionnaire [14]. Nurse AMIE read the questions to the participant. The system has built in skip logic, so questions only appeared if they were required based on the previous answer. Once the participants completed the study, the Nurse AMIE skill was disabled and removed from the participants’ device, and they no longer received messages/interventions from Nurse AMIE.

### 2.15 Outcomes

Patient acceptability was defined as the percentage of eligible and approached participants agreeing to participate in the intervention. We observed an acceptability of 68% with the tablet-based Nurse AMIE intervention, and we hypothesized the Nurse AMIE for Amazon Echo Show version would have a comparable or higher acceptability rate [8]. We defined feasibility the same as the tablet-based Nurse AMIE intervention: participating in at least 30 of the first 90 days. We observed that 86% of our participants in our prior studies that used a tablet version of the intervention achieved this definition of feasibility. We anticipate it would be equally high if not higher with the Amazon Echo Show version.

Patient reported outcomes measures included the 36-Item Short Form Survey (SF-36), the Brief Pain Inventory (BPI), Brief Fatigue Inventory (BFI), PROMIS sleep disturbance measure (PROMIS SF v1.0 - Sleep Disturbance 8b), and a measure of distress used at the Penn State Cancer Institute [[15], [16], [17], [18]]. Outcomes were assessed at baseline, the end of month three, and end of month six. Additionally, assessments related to the usability of Nurse AMIE were collected at the end of month three and six, and included: a User Version of the Mobile Application Rating Scale (U-MARS), System Usability Scale (SUS), Client Satisfaction Questionnaire (CSQ-8), Credibility and Expectancy Questionnaire (CEQ), and Technology Acceptance Model (TAM) [[19], [20], [21], [22]]. Participants completed these outcome questionnaires over Zoom.

### 2.16 Sample size

Based on previous data from the tablet version of Nurse AMIE, the standard deviation for physical distress in MBC patients at baseline was 1.9 units, on a scale of 0–10, and the change over three months was 2.7. To be conservative, we consider the difference of outcome change over three months between two groups as 1.8 (effect size = 0.95). Using a power level of 0.80, and setting (two-sided) α at 0.05, 18 participants per group provided sufficient power to detect the expected between-group difference. Given the potential for disease progression that would prevent continued participation in this population, we anticipated a loss to follow-up of approximately 15%. As such, we recruited 21 participants per group.

### 2.17 Data and safety monitoring

The study facilitators reviewed the daily symptom data on the dashboard daily. Adverse events were formally assessed monthly, using the National Institute of Heath developed PRO-CTCAE measure [23]. In the unlikely event an unanticipated adverse event were deemed to be definitely related to the study occurred, the relative merits and risks of continuing the research would be discussed with the treating physician. The Data Safety Monitoring Committee of the Cancer Institute would be informed of any such actions, but none occurred.

### 2.18 Statistical analyses

Data analyses will be performed using SAS version 9.4. Summary statistics including mean and standard deviations for continuous variables and frequency with percentage for categorical variables are reported. The normality assumption for outcome variables is assessed by group based on Shapiro Wilk tests, and the skewness and potential outliers of data distribution will be evaluated to see if any transformation will be needed. Between group (immediate AMIE baseline to month three, and delayed AMIE month four to six) comparison will serve as the primary analyses, where two-sample *t*-tests or Wilcoxon rank sum tests will be used, as appropriate. To further provide evidence informing potential future studies, or may point towards temporal trends or variability in each group, changes within-group (baseline to month three, month four to six) will also be explored mainly with summary statistics. Generalized linear mixed effect models for group comparison will be fitted, and the variables of age, cancer treatment type, and cancer treatment duration will be initially examined and controlled. If we detect some baseline characteristic variables showing unbalanced between two groups, we will consider them in the regression, and report them. The amount of missing data will be reported, which is expected to be minimal due to previous data collection experience. We will try best to collect the data, and report the number of missing data and mechanisms for this missing data (e.g., loss to follow-up, technical errors). If any missing data exist, the missing mechanism will be evaluated. Based on previous work and previous versions of this trial on the tablet, it is reasonable to assume that data will be missing at random (MAR), under which the likelihood based analyses will yield valid estimates and valid inference.

## 3 Discussion

This pilot study, building on the previously successful tablet-based Nurse AMIE, will examine the feasibly, acceptability, usability and benefits of a supportive health care platform conducted on the Amazon Echo Show. This study will also report a wealth of relevant information related to the use of varying technology to intervene on MBC patients, including enjoyment, engagement, satisfaction, and willingness to recommend the device to friends and other MBC patients. Findings from the study will also be used to develop future Nurse AMIE interventions; future Nurse AMIE interventions will be expanded to provide additional content within the platform, use emerging technology to more effectively intervene on MBC patients, and provide Nurse AMIE interventions to under-represented/under-studied populations in a cost-effective and scalable manner.

The novelty of this intervention is a strength. The technological capabilities of the Amazon Echo Show allowed the use of voice to communicate with the device, which may be a simpler and more accessible means than other methods, such as touch-screens, smart watches, and websites. Another strength of the trial is the lack of requiring in-person assessments. Interventions to address quality of life in MBC patients requiring study-related visits have reported low adherence; the ability to conduct this trial fully remotely is a strength [24].

Limitations include a comparatively small sample size, and the requirement that individuals participating in the intervention had access to Wi-Fi, and a digital device capable of receiving Zoom calls. Future work should focus on incorporating these modalities to support MBC patients in populations and areas with less access to cancer care, and less access to technology such as Wi-Fi, to limit accentuating existing disparities in cancer care [25].

In conclusion, technology may be useful for enhancing patient-provider communication, and reducing symptom burden in MBC patients. With novel technologies rapidly advancing, the best way to use technology to intervene on MBC patients is unclear. We piloted Nurse AMIE, a digital supportive care platform that provides guideline-concordant symptom management interventions in response to patient reported outcomes, among MBC patients using an Amazon Echo Show device. Findings will inform technological and trial-related factors that influence the feasibility, acceptability, enjoyment, and effects of these interventions on patient reported outcomes.

## Funding

American Institute for Cancer Research.

## Author contributions

All authors were involved in the conceptualization, writing, review, and editing. All authors have given final approval of the version to be published.

## Declaration of competing interest

The authors declare that they have no known competing financial interests or personal relationships that could have appeared to influence the work reported in this paper.

## Data Availability

No data was used for the research described in the article.
